# Short term and intermediate results of hybrid procedure in high risk patients needing aortic arch repair

**DOI:** 10.1186/1749-8090-8-S1-O45

**Published:** 2013-09-11

**Authors:** JH Yang, JB KIM, SH Jung, SJ Choo, JW Lee, CH Chung

**Affiliations:** 1 Department of Thoracic and Cardiovascular Surgery, Asan Medical Center, University of Ulsan College of Medicine, Seoul, Korea

## Background

Hybrid procedures for aortic arch and proximal descending thoracic aorta repair are becoming popular as an alternative management for high risk patients with comorbidities. This article is aimed to assess the short term and midterm outcome of hybrid procedures in aortic arch and descending thoracic aortic repair.

## Methods

From December 1, 2007 through February 28, 2013, 44 patients who underwent hybrid procedures for aortic arch and proximal descending aorta repair were retrospectively reviewed for incidence of complications and early mortalities. Of the 44 patients, Ishimaru zone 0 deployment was proceeded in 24 cases, zone 1 deployment in 4 and zone 2 deployment in 16.

## Results

Patients’ mean age was 64.9 years (21~85). Perioperative neurologic complication occurred in 7 (16.0%) patients, endoleak needing extra procedure occurred in 4 (9.1%), stent related complication other than endoleak occurred in 2 (4.5%), which were stent stenosis and left common carotid artery occlusion by stent. Iatrogenic AR occurred in 1 (2.3%), whom AVR was operated on. Newly developed AF occurred in 2 (4.5%). Mediastinitis occurred in one case (2.3%). There was one early mortality (2.3%). The cause was refractory VF (ventricular fibrillation). 12 all-cause late mortalities occurred (27.3%).

## Conclusions

Compared with open arch surgeries which were performed at the same institute, during same period, hybrid procedures did not show inferior results. Considering that the patients who underwent hybrid procedures were much older and had more risk factors, we may suggest that hybrid procedures can be reasonable alternative methods for those who have high risks and contraindicated for open surgery.

